# OptiPrep? Density Gradient Solutions for Macromolecules and Macromolecular Complexes

**DOI:** 10.1100/tsw.2002.844

**Published:** 2002-06-07

**Authors:** John Graham

**Affiliations:** School of Biomolecular Sciences, Liverpool John Moores University, UK

**Keywords:** gradient solutions, OptiPrep™, iodixanol, density, osmolality, pH, mammalian cells

## Abstract

Any density gradient for the isolation of mammalian cells should ideally only expose the sedimenting particles to an increasing concentration of the gradient solute. Thus they will experience only an increasing density and viscosity, other parameters such as osmolality, pH, ionic strength and the concentration of important additives (such as EDTA or divalent cations) should remain as close to constant as possible. This Protocol Article describes the strategies for the dilution of OptiPrep™ in order to prepare such solutions for mammalian cells.

**METHOD TYPE:** extraction, isolation, purification and separation

**SUB METHOD TYPE:** centrifugation

## OSMOLALITY

The observed osmolality of OptiPrep™ depends on the mode of measurement (freezing point or vapour pressure). The former provides a value of approx. 260 mOsm, the latter approx. 170 mOsm. Moreover, irregular freezing of the sample sometimes makes measurement of the freezing point difficult. However, when OptiPrep™ is diluted with water to 50% iodixanol, values of approx. 195 mOsm are consistently obtained. More importantly, OptiPrep™ can be diluted with a buffered isoosmotic solution to provide a working solution that is also isoosmotic.

## pH

Because it is a solution in water with no additives, its pH (4-6) is that of water. The actual pH is unimportant, because when OptiPrep™ is mixed with a standard ionic or organic buffer, the pH of the buffer is unaffected by the iodixanol: any changes in pH are the same as those that would occur during dilution of the buffer with water.

## HANDLING OptiPrep™

Prolonged exposure (several months) of iodixanol solutions to sunlight will cause a slow release of iodine; OptiPrep™ should therefore be stored away from strong sunlight. On standing, iodixanol may "settle out" of concentrated solutions; always shake the bottle of OptiPrep™ before use.

## PREPARATION OF DENSITY SOLUTIONS

Macromolecules and macromolecular complexes cover such a diverse range of particles that it is not possible to give generic recipes for the preparation of gradient solutions from OptiPrep™. Instead, some of the most commonly used media which have been used as solvents for gradient solutes for the purification of viruses, nucleic acids, proteins, nucleoprotein particles, and lipoproteins are listed with indications as to the manner in which they may be prepared with OptiPrep™.

## GENERAL STRATEGIES

If it is important to maintain the concentration of a particular buffer or reagent constant throughout the gradient, then the general strategy is as follows.

Make a 50% (w/v) iodixanol working solution by diluting 5 vol of OptiPrep™ with a 1 vol of a diluent containing 6x the required concentrations of reagents.

The working solution will contain the correct concentration of reagents; this can then be further diluted with the normal medium to provide solutions of lower density. The density of the working solution will depend on the density of the diluent. This working solution can also be added directly to a sample in order to adjust its density.

Macromolecules and macromolecular complexes traditionally have been purified in gradients containing high concentrations of sucrose, glycerol, alkali metal salts (e.g., KBr and NaCl) or heavy metal salts (e.g., CsCl). The particles have therefore been isolated in grossly hyperosmotic conditions. OptiPrep™ offers the opportunity to isolate them under isoosmotic conditions. Depending on the concentration of the reagents it may be necessary to include in ALL diluents the same concentration of an osmotic balancer such as 0.85% NaCl or 0.25 M sucrose[1,2].

In many cases it may be sufficient simply to dilute the OptiPrep™ with a medium containing the reagents at their normal concentrations, in which case any iodixanol gradient will also contain an inverse gradient of the reagents.

## VIRUSES

Gradient media for these particles are commonly prepared by dilution of OptiPrep™, with a buffered saline solution. The buffer may be organic (e.g., Hepes-NaOH) or phosphate. Sometimes low concentrations of KCl (2.5 mM) or $MgCl_2$ (1 mM) may be included.

## DNA/RNA

Gradients containing 1 mM EDTA, 10 mM NaCl, 10 mM Tris-HCl, pH 7.5 are not uncommon. See Ref. [3] for more information of the effect of gradient composition on the banding density of nucleic acids in iodinated density gradient media.

## PROTEINS

Soluble proteins have been banded in gradients produced by dilution of OptiPrep™ with a simple Hepes-buffered saline, but often other reagents that may stabilize the protein are included. Basi and Rebois[4], for example, included 20 mM Hepes-NaOH, pH 8.0, 1 mM EDTA, 1 mM DTT, 2 mM $MgSO_4$, and 0.1% Lubrol PX in iodixanol gradients for studying the sedimentation of proteins. At the concentrations used, these reagents will have little effect on the density or osmolality of the gradient.

## NUCLEOPROTEINS AND NUCLEOPROTEIN COMPLEXES

The banding of DNA-protein complexes usually occurs in gradients containing salt since the complexes are unstable in its absence(e.g., 0.14 M NaCl, 1 mM DTT, 0.1 mM EDTA, 10 mM Tris-HCl, pH 7.5). For more information see Ref. [3]. There is a wide variety of (often complex) media that are used in gradients for the isolation of ribonucleoproteins and there are several excellent reviews on the fractionation of these complexes in a variety of media[5,6,7] which give details of the required gradient composition.

## LIPOPROTEINS

Simple dilutions of OptiPrep™ with Hepes-buffered saline suffice for the fractionation of lipoproteins and antioxidants may be included at the discretion of the operator.

The density of any gradient solution can be calculated using Eq. 1, so long as the densities of the iodixanol-containing solution and of the diluents are known.

$$D = \frac{Vd + V_1 d_1}{V + V_1} \tag{1}$$

$D$ = Density of mixture; $V$ = volume of OptiPrep™; $d$ = density of OptiPrep™; $V_1$ = volume of diluent; $d_1$ = density of diluent

## ACKNOWLEDGEMENTS

The author and TheScientificWorld wish to thank Axis-Shield PoC, AS, Oslo, Norway for their kind permission to adapt OptiPrep™ Application Sheet M1 in the preparation of this Protocol Article.
